# Evaluation of the Antidiarrheal Activity of 80% Methanolic Extract and Solvent Fractions of *Calpurnia aurea* (Ait.) Benth. (Fabaceae) Roots in Swiss Albino Mice

**DOI:** 10.1155/bmri/2594439

**Published:** 2026-08-29

**Authors:** Yared Andargie Ferede, Mulugeta Molla Zeleke, Tilaye Arega Moges, Achenef Bogale Kassie, Woretaw Sisay Zewdu, Fikadie Dagnew Baye, Muluken Adela Alemu

**Affiliations:** ^1^ Pharmacology Unit, Department of Pharmacy, College of Health Sciences, Debre Tabor University, Debre Tabor, Ethiopia, dtu.edu.et; ^2^ Clinical Pharmacy Unit, Department of Pharmacy, College of Health Sciences, Debre Tabor University, Debre Tabor, Ethiopia, dtu.edu.et; ^3^ Department of Pediatric and Child Health Nursing, College of Medicine and Health Sciences, Bahir Dar University, Bahir Dar, Ethiopia, bdu.edu.et

**Keywords:** 80% methanol, antidiarrheal, *Calpurnia aurea*, mice, root, solvent fractions

## Abstract

**Introduction:**

In Ethiopian traditional medicine, the roots of *C. aurea* have been used to treat severe diarrhea. This study is aimed at scientifically validating the antidiarrheal activity of 80% methanol *C. aurea* root extract and solvent fractions in mice.

**Methods:**

Qualitative phytochemical analysis was performed, and the acute toxicity study was conducted following OECD guideline 423. The antidiarrheal efficacy of the crude extract and solvent fractions was evaluated using castor oil–induced diarrhea, enteropooling, and intestinal motility models. Mice were randomly assigned to five groups: Group I received distilled water (10 mL/kg) and served as the negative control; Group II received either loperamide (3 mg/kg) or atropine sulfate (5 mg/kg) as the positive control; and Groups III–V received 100, 200, and 400 mg/kg of the crude extract or its solvent fractions, respectively. All treatment effects were compared to the negative control.

**Results:**

The acute toxicity study confirmed that the crude extract was safe at a limit test dose of 2000 mg/kg. Phytochemical screening test revealed the presence of flavonoids, phenols, tannins, saponins, terpenoids, and alkaloids. In the castor oil–induced diarrhea model, the crude extract at 200 mg/kg (*p* < 0.01) and 400 mg/kg (*p* < 0.001) significantly delayed the onset of diarrhea and reduced wet feces. At these test doses, the extract also markedly (*p* < 0.01) decreased the weight and volume of intestinal contents in the enteropooling test and reduced the peristaltic index (*p* < 0.01) in the charcoal meal test. Among the solvent fractions, the aqueous fraction produced substantial dose‐dependent activity (*p* < 0.05) at all test doses across all models, with the highest effect at 400 mg/kg (*p* < 0.001). The ethyl acetate fraction also showed notable effects at 200 mg/kg (*p* < 0.05) and 400 mg/kg (*p* < 0.01), whereas the chloroform fraction was active only at 400 mg/kg (*p* < 0.05).

**Conclusion:**

The crude extract and solvent fractions of *C. aurea* roots exhibited statistically significant, dose‐dependent antidiarrheal activity. Therefore, these findings support the traditional use of this plant for treating diarrhea.

## 1. Introduction

Diarrhea is commonly defined as the frequent passage of loose or unformed stools, typically occurring more than three times a day [1], and is characterized by the excretion of over 200 g of stool daily [2]. Globally, diarrheal diseases represent a critical public health issue, particularly affecting children under 5 years old [3]. This condition accounts for approximately 9% of global mortality, making it the second leading cause of death after respiratory tract infections [4]. Although diarrheal disease occurs globally, its prevalence is highest in low‐income countries, especially in sub‐Saharan Africa and South Asia [5]. Despite advances in interventions, diarrhea continues to pose a serious threat to life in these areas [6]. In Ethiopia, it ranks as the second leading cause of death after pneumonia, claiming the lives of nearly half a million children under the age of 5 each year [7]. Pathologically, diarrhea results from an imbalance between fluid secretion and absorption in the intestinal lumen [8]. Based on its underlying mechanisms, diarrhea is classified into secretory, osmotic, inflammatory, and motility‐related diarrhea [8].

Diarrhea is typically alleviated through the use of either conventional antidiarrheal agents or herbal remedies [9]. The former are well‐established for their effectiveness in stabilizing stool consistency, reducing the frequency of bowel movements, and decreasing stool volume [10]. These effects result from various mechanisms, including inhibition of intestinal fluid secretions, modulation of intestinal motility, and enhancing absorption of the intestinal lumen [11].

Current antidiarrheal agents remain inaccessible to many people, particularly in developing countries, and are further limited by significant drawbacks. Oral rehydration salts (ORS) and zinc supplementation prevent dehydration and reduce recurrence but do not directly control stool output [12]. Loperamide and related agents may cause adverse effects, including constipation, paralytic ileus, or toxic megacolon, and are not advised for infectious diarrhea or use in children [13]. Moreover, the administration of antibiotics such as ciprofloxacin and co‐trimoxazole contributes to superinfections and accelerates antimicrobial resistance [14]. In contrast, traditional remedies are widely available, affordable, and culturally acceptable to large segments of the population [15]. As a result, a significant portion of the population widely practices them as a preferred therapeutic option, especially in low‐income countries [16].

According to reports from the World Health Organization (WHO), a large number of individuals in resource‐limited countries primarily depend on herbal products to meet their healthcare needs. Particularly in rural areas, the consumption rate is high, accounting for 80% of the population [17]. Similarly, in Ethiopia, about 80%–90% of the population uses herbal agents as a major therapeutic option for managing various ailments [15]. In the past 30 years, almost 50% of all modern pharmaceuticals have been developed from natural products or their derivatives [18]. For instance, crofelemer, a compound extracted from the red latex of the *Croton lechleri* tree, has shown promise in treating chronic idiopathic diarrhea [19]. Likewise, berberine, an isoquinoline alkaloid derived from *Rhizoma coptidis* and *Rhizoma phellodendri*, has been used as an antidiarrheal agent for centuries in China [20]. Collectively, all this evidence highlights the need to explore safe, effective, and accessible herbal antidiarrheal drugs derived from natural products, particularly from *C. aurea.*

*C. aurea* is a flowering plant belonging to the Fabaceae family. This genus includes shrubs and small trees typically found at the edges of forests across East Africa, including Ethiopia, as well as in Southern India [21]. In Ethiopia, it is known by various vernacular names, such as “digita” in Amharic and “chekata” in Afan Oromo [22]. Previous pharmacological investigations of *C. aurea* have primarily focused on its leaves and seeds, demonstrating a range of biological activities including antimicrobial and antidiarrheal effects. Furthermore, in vitro studies of the root extract have reported antibacterial and antioxidant properties [23–26]. However, despite the widespread use of *C. aurea* roots in Ethiopian traditional medicine for the management of diarrhea [27], their in vivo antidiarrheal activity remains unexplored. This represents an important knowledge gap, as the composition and abundance of bioactive phytochemicals often vary among plant organs, preventing the extrapolation of findings from the leaves or seeds to the roots. To address this gap and provide scientific support for its traditional use, the present study evaluated the in vivo antidiarrheal activity of the 80% methanolic root extract and its solvent fractions using established murine models. Accordingly, the study complements previous investigations of the leaves and seeds and broadens the experimental evidence supporting the antidiarrheal potential of *C. aurea*.

## 2. Materials and Methods

### 2.1. Drugs, Chemicals, and Instruments

The laboratory‐grade drugs, chemicals, and instruments listed below were used in this study: Absolute methanol (Glenmark Pharmaceuticals, India), atropine sulfate (White Swan Pharmaceuticals, India), loperamide hydrochloride (Vasudha Pharma Chem. Ltd, India), ketamine hydrochloride (Neon Laboratories Ltd, India), xylazine (Neon Laboratories Ltd, India), castor oil (Amman Pharmaceutical Industries, Jordan), distilled water (Debre Tabor Comprehensive Specialized Hospital, Ethiopia), chloroform (Carlo Erba Reagents, France), ethyl acetate (Laxmi Organic Industries Ltd, India), activated charcoal (Zerfam International Business PLC, Ethiopia), Mayer′s and Dragendorff′s reagents (May and Baker Ltd, Dagenham, England), ferric chloride (BDH Ltd, England), potassium ferrocyanide (BDH Ltd, England), ammonia (Merck Millipore, India), lead acetate reagent (Fisher Scientific, United Kingdom), sulfuric acid (Farm Italia Carlo Erba, Italy), mini orbital shaker (Spectralab Instruments Pvt. Ltd, India), rotary evaporator (Henan Lanphan Industry Co. Ltd, China), lyophilizer (Labtop Instruments Pvt. Ltd, India), refrigerator (Meling Biomedical, China), and separatory funnel (IWAKI Glass Co. Ltd, Japan).

### 2.2. Collection and Identification of Plant Material

Fresh and healthy root samples of *C. aurea* were collected in November 2024 from the Libo Kemkem district, about 100 km from Bahir Dar (capital of the Amhara region) and 665 km northwest of Addis Ababa, Ethiopia. Collection was carried out on private land with the landowner′s permission. Because the species is not protected, no government license was required. The plant was authenticated by Dr. Achenef Belay, Department of Biology, Debre Tabor University, through comparison with reference specimens, and a voucher specimen (AY131) was deposited in the university herbarium. After approval, bulk quantity of roots was collected and rinsed with cold water, air‐dried at room temperature, pulverized into fine powder using an electric grinder, and stored in an airtight container until extraction.

### 2.3. Preparation of Crude Extract and Fractionation

#### 2.3.1. Crude Extract Preparation

**Figure 1:**
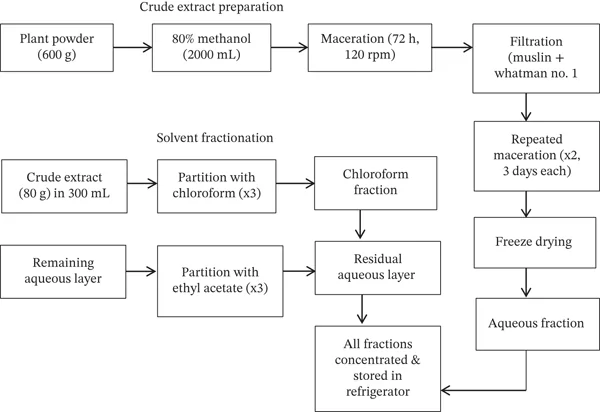
Diagrammatic representation of the crude extract preparation and solvent fractionation of *C. aurea* root.

A total of 600 g of coarse plant powder were immersed in 2000 mL of solvent, prepared by mixing 1800 mL of methanol with 200 mL of distilled water (3:10 w/v), in a sealed, large conical flask. The mixture was then subjected to maceration at room temperature for 72 h, with intermittent agitation using a miniorbital shaker set at 120 rpm. The extract was filtered through muslin cloth twice followed by Whatman No. 1 filter paper. The residue was remacerated twice with fresh 80% methanol (each for 72 h) using the same procedure and the filtrates were pooled. The combined extract was concentrated under reduced pressure at 40°C–45°C using a rotary evaporator, then freeze‐dried to remove residual moisture. The percentage yield was calculated, and the dried extract was stored in labeled amber glass containers at 4°C until further use (Figure 1).

#### 2.3.2. Preparations of Solvent Fractions

The crude extract of *C. aurea* (80 g) was further fractionated using chloroform, ethyl acetate, and distilled water in order of increasing polarity, following a modified method by Ayal et al. [28]. The extract was dissolved in 300 mL of distilled water and successively partitioned with equal volumes of chloroform and ethyl acetate. Each solvent extraction was performed twice, and the combined organic layers were concentrated under reduced pressure to yield the chloroform fraction (CF) and ethyl acetate fraction (EF). The residual aqueous layer was concentrated to obtain the aqueous fraction (AF). Each fraction was weighed, and the percentage yield was calculated before storage in airtight containers at 4°C until further use (Figure 1). The yield was calculated using the following formula:

(1) %yield=Weight of the extractWeight of the total sample×100.

### 2.4. Qualitative Phytochemical Evaluation

The crude extracts and solvent fractions of the root of *C. aurea* were qualitatively screened for the presence of secondary metabolites such as flavonoids, tannins, terpenoids, saponins, phenols, steroids, glycosides, alkaloids, and anthraquinones using internationally accepted protocols [29].

### 2.5. Acute Toxicity Study

The acute toxicity test of the crude extract was conducted following Organization for Economic Co‐operation and Development (OECD) guideline 423 [30]. Healthy adult female Swiss albino mice (20–30 g) were used, as females are generally more sensitive to toxic compounds. The animals were housed under standard laboratory conditions and acclimated for 7 days. Mice were randomly assigned to groups of three and fasted overnight before dosing. A single limit dose of 2000 mg/kg body weight was administered orally via gavage to the first group, and the animals were closely observed for the first 30 min, periodically for 4 h, and daily for 14 days. If no mortality occurred, a second group received the same dose; if mortality occurred, a lower dose (300 mg/kg) was tested following a stepwise approach. Animals were monitored for mortality and signs of toxicity, including reduced food and water intake, weight loss, and behavioral, neurological, or physical changes.

### 2.6. Experimental Animals

Healthy adult Swiss albino mice (both sexes), weighing 25–30 g and aged 8–12 weeks, were used in the study. The animals were obtained from the animal house of the Department of Pharmacology, Debre Tabor University, and housed in polypropylene cages under standard laboratory conditions (22^°^C ± 2^°^C, 55*%* ± 10*%*relative humidity, 12‐h light/dark cycle). They were provided with a standard pellet diet and water ad libitum and acclimated to the laboratory environment for 7 days before the experiment. All procedures were conducted in accordance with the Animal Research Reporting of In Vivo Experiments (ARRIVE) guidelines to ensure proper care and use of laboratory animals.

### 2.7. Animal Grouping, Dosing, and Experimental Bias Control

In all three experimental models, healthy adult Swiss albino mice of both sexes (20–30 g) were randomly assigned to five groups (*n* = 6 per group). This sample size was selected based on existing studies evaluating antidiarrheal activity, balancing statistical reliability with animal reduction in accordance with the principles of replacement, reduction, and refinement (3Rs). To prevent baseline bias, animals were stratified by body weight prior to allocation using a computer‐generated random sequence, ensuring equal mean weights across groups.

Prior to testing, mice were fasted for 18 h with free access to water. Group I (negative control) received distilled water (10 mL/kg), whereas Group II (positive control) received reference drugs: loperamide (3 mg/kg) for the castor oil–induced diarrhea and enteropooling models, or atropine sulfate (5 mg/kg) for the gastrointestinal motility assay [31]. Groups III–V (test groups) received the crude extract or solvent fractions at 100, 200, and 400 mg/kg, respectively. Doses were selected based on an acute oral toxicity study demonstrating safety up to 2000 mg/kg [6]; the median dose was set at 1/10th of this limit (200 mg/kg), with low (100 mg/kg) and high (400 mg/kg) doses set at half and twice the median dose, respectively. Doses for solvent fractions were aligned with the crude extract, consistent with a previous study [32].

To minimize experimental bias throughout the study, all animals were maintained under identical environmental conditions and handled using standardized procedures. Treatments were prepared, coded, and administered by an independent assistant not involved in data collection. Outcome assessments, data collection, and statistical analyses were performed by investigators blinded to treatment allocations using predefined evaluation criteria.

### 2.8. Determination of Antidiarrheal Activity

#### 2.8.1. Castor Oil–Induced Diarrheal Model

A total of 84 mice (both sexes) were fasted for 18 h, randomly assigned to groups, and treated as described previously. The castor oil–induced diarrhea model was performed following Megersa et al. [2]. At 1 h after treatment, each mouse received 0.5 mL of castor oil orally via gavage and was individually housed in a metabolic cage lined with white paper for clear visualization and accurate fecal quantification. The paper was replaced after each defecation. During the 4‐h observation period, the onset of defecation, number and weight of wet stools, and total fecal output were recorded and compared to the control group. The percentages of diarrheal inhibition, wet, and total fecal output were then calculated using the formula below:

(2) %inhibition of defecation=Mean number of we feces NC−TGMean number of wet defecation in NC×100,

(3) %of wet fecal output=Mean weight of wet feces of each TGMean weight of wet feces of the NC×100,

(4) %of total fecal output=Mean weight of fecal output in each TGMean weight of fecal output in the NC×100,

where NC is the negative control and TG is the test group.

#### 2.8.2. Castor Oil–Induced Enteropooling Test

With minor modifications, the procedure described by Gudeta et al. [33] was employed to evaluate the effect of the test extract on intestinal contents. After an 18‐h fasting, 84 mice received treatments as previously described. After 1 h, diarrhea was induced with 0.5 mL of castor oil orally. The mice were anesthetized with ketamine (80 mg/kg) and xylazine (5 mg/kg) and euthanized via cervical dislocation following ethical guidelines. The small intestine was ligated at the pyloric and cecal ends, excised, weighed, and its contents expressed into a graduated cylinder to measure volume. The emptied intestine was reweighed, and the difference in weight calculated. The percentage inhibition of intestinal secretion was then determined using the following formula:

(5) %inhibition=A−BA×100,

where A denotes the average volume or weight of the intestine in the control group, whereas B denotes the average volume or weight of the intestine in the test groups.

#### 2.8.3. Castor Oil–Induced Charcoal Meal Test

The antimotility effect of the extract was evaluated using the method described by Zewdie et al. [34]. Male and female mice were fasted for 18 h with free access to water and treated as described in Section 2.7. After 1 h, diarrhea was induced with 0.5 mL of castor oil orally, followed by 1 mL of 5% charcoal suspension after another hour. The mice were then anesthetized with ketamine (80 mg/kg) and xylazine (5 mg/kg) and euthanized via cervical dislocation in accordance with ethical guidelines. The small intestine was excised, and the distance traveled by the charcoal marker from the pylorus to the cecum was measured and expressed as a percentage of the total intestinal length. The peristalsis index and percentage inhibition were calculated using the standard formula below:

(6) Peristaltic index=Mean distance travelled by charcoal mealMean length of small intestine×100,

(7) %inhibition of motility=PIC−PIT PIC×100,

where PIC is the peristaltic index of the control and PIT is the peristaltic index of the test group.

#### 2.8.4. In Vivo Antidiarrheal Index (ADI)

The ADI of the crude extract, solvent fractions, and standard drug was calculated by integrating three parameters from the aforementioned models using the formula presented [35]:

(8) In vivo ADI=Dfreq x Gmeq x Pfreq3,

where Dfreq is the delay in defecation time (% of control), Gmeq is the reduction in gut meal travel (% of control), and Pfreq is the purging frequency or reduction in the number of feces (% of control). Each parameter was calculated using the following formulas:

(9) Dfreq=Mean onset of diarrhea of the test−control groupMean onset of diarrhea of the control group×100,

(10) Gmeq= Distance travelled by the charcoal marker of the control−test groupDistance travelled by the charcoal marker in the control group×100,

(11) Pfreq=Mean number of wet feces of control−testMean number of the wet feces of the control group×100.

### 2.9. Quality Control

Data reliability was ensured through randomization and blinding, adherence to standardized protocols, the use of validated animal models, proper statistical analysis, and the deployment of analytically graded materials.

### 2.10. Statistical Analysis

Data were expressed as mean ± standard error of the mean (SEM). Prior to analysis, the assumptions of one‐way ANOVA were formally assessed using the Shapiro–Wilk test for normality and Levene′s test for homogeneity of variances. As these assumptions were satisfied (*p* > 0.05), comparisons among groups were performed using one‐way ANOVA followed by Tukey′s post hoc multiple comparison test using SPSS Version 26. A 95% confidence level was applied, and *p* values < 0.05 were considered statistically significant.

## 3. Results

### 3.1. Extraction Yield of the Crude Extract and Solvent Fractions

The extraction yields of the crude extract and its solvent fractions are shown in Table 1. The extraction process yielded 99.6 g of crude extract (16.6%). Of this, 80 g was subjected to solvent fractionation, resulting in percentage yields of 24.6%, 33.0%, and 42.4% for the CF, EF, and AF, respectively.

**Table 1:** Percentage yield of the crude extract and solvent fractions of the roots of *C. aurea*.

Type of extract	Weight obtained (g)	Percentage yield (%)
Crude extract	99.6	16.6
Chloroform fraction	19.7	24.6
Ethyl acetate fraction	26.4	33.0
Aqueous fraction	33.9	42.4

Abbreviations: %, percent; g, gram.

### 3.2. Phytochemical Screening Test

Phytochemical screening of the crude extract and solvent fractions from *C. aurea* roots revealed the secondary metabolites listed in Table 2.

**Table 2:** Phytochemical analysis of the crude extract and solvent fractions of the root of *C. aurea*.

Screened phytochemicals	Crude extract	CF	EF	AF
Phenols	+	−	+	+
Flavonoids	+	−	−	+
Terpenoids	+	−	+	+
Tannins	+	−	−	+
Saponins	+	−	+	+
Alkaloids	+	+	+	+
Steroids	+	+	+	−
Glycosides	−	−	−	−
Anthraquinones	−	−	−	−

*Note:* (+) means present and (−) means absent.

Abbreviations: AF, aqueous fraction; CF, chloroform fraction; EF, ethyl acetate fraction.

### 3.3. Acute Oral Toxicity Study

During the 14‐day follow‐up, no mortality or signs of toxicity were observed. Food and water intake, body weight, and behavioral or physical parameters remained normal in all animals. These results indicate that the crude extract of the root of *C. aurea* is well tolerated at the tested dose, supporting its classification as orally nontoxic according to OECD guideline 423.

### 3.4. Determination of Antidiarrheal Activity

#### 3.4.1. Castor Oil–Induced Diarrhea

**Figure 2:**
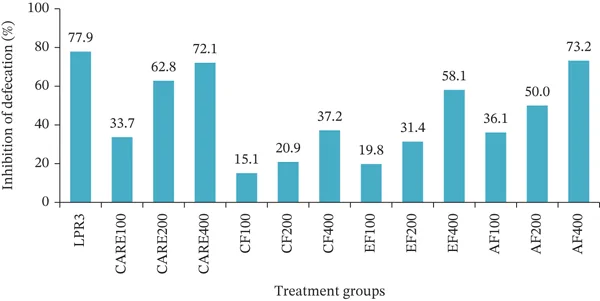
Percentage inhibition of castor oil‐induced diarrhea by *C. aurea* crude extract and solvent fractions in mice. Note: Mice were orally pretreated with vehicle (distilled water, 10 mL/kg), loperamide (LPR3; 3 mg/kg; positive control), the 80% methanolic crude extract of *C. aurea* (CARE; 100, 200, and 400 mg/kg), or the chloroform fraction (CF), ethyl acetate fraction (EF), and aqueous fraction (AF) at doses of 100, 200, and 400 mg/kg (*n* = 6 mice per group). Percentage inhibition of diarrhea was calculated relative to the negative control group.

Among the crude extract‐treated groups, the *C. aurea* root extract 400 mg/kg dose (CARE400) showed the most significant effect (*p* < 0.001), delaying diarrhea onset and reducing the number and weight of wet feces. The *C. aurea* root extract 100 mg/kg (CARE100) and the *C. aurea* root extract 200 mg/kg (CARE200) doses also exhibited dose‐dependent effects, with only CARE200 producing a significant delay in diarrhea onset (*p* < 0.01) and reduction in the number and weight of wet feces (*p* < 0.05). Among the solvent fractions, the AF showed significant activity (*p* < 0.05) at all tested doses, with the greatest effect at 400 mg/kg (*p* < 0.001). At this test dose, the maximum inhibition of diarrhea (73.25%) was achieved, comparable to that of loperamide (Figure 2). The EF demonstrated antidiarrheal activity at 200 and 400 mg/kg, with the highest dose significantly delaying diarrhea onset (*p* < 0.01) and reducing the number and weight of wet feces (*p* < 0.01). The CF was active only at 400 mg/kg for all parameters (*p* < 0.01) (Table 3).

**Table 3:** Effects of the crude extract and solvent fractions of the root of *C. aurea* on castor oil–induced diarrhea in mice.

Group	Onset of diarrhea (min)	No of wet feces	Total no of feces	Wt. of wet feces	Wt. of total feces	% inhi. of defecation	%WWFO	%WTFO
NC	40.6 ± 2.3	8.6 ± 2.1	10.1 ± 2.8	3.8 ± 1.0	4.3 ± 2.2	—	—	—
LPR3	124.2 ± 2.8^a^ ^∗∗∗^	1.9 ± 0.9^a^ ^∗∗∗^	2.7 ± 1.1^a^ ^∗∗∗^	0.8 ± 0.5^a^ ^∗∗∗^	1.0 ± 1.0^a^ ^∗∗∗^	77.9	21.1	23.3
CARE100	54.4 ± 4.7	5.7 ± 1.4	7.0 ± 3.0	3.3 ± 0.9	3.9 ± 2.5	33.7	86.8	90.7
CARE200	90.6 ± 5.9^a^ ^∗∗∗^	3.2 ± 2.7^a^ ^∗∗^	4.3 ± 2.5^a^ ^∗∗^	2.0 ± 1.2^a^ ^∗^	2.4 ± 1.9^a^ ^∗^	62.8	52.6	55.8
CARE400	114.7 ± 2.6^a^ ^∗∗∗^	2.4 ± 0.8^a^ ^∗∗^	3.1 ± 1.3^a^ ^∗∗∗^	1.4 ± 1.5^a^ ^∗∗^	1.6 ± 0.9^a^ ^∗∗^	72.1	36.8	37.2
CF100	50.8 ± 2.9	7.3 ± 1.9	9.2 ± 3.7	3.5 ± 2.0	4.1 ± 2.3	15.1	92.1	95.3
CF200	56.2 ± 3.4	6.8 ± 2.3	8.9 ± 2.7	3.3 ± 1.7	3.8 ± 1.8	20.9	86.8	88.4
CF400	67.4 ± 4.2^a^ ^∗∗^	5.4 ± 1.4^a^ ^∗∗^	6.6 ± 2.0^a^ ^∗∗^	2.7 ± 1.3^a^ ^∗∗^	2.9 ± 2.0^a^ ^∗^	37.2	71.1	67.4
EF100	52.5 ± 3.7	6.9 ± 3.2	8.4 ± 1.8	3.4 ± 2.4	4.0 ± 1.6	19.8	89.5	93.0
EF200	70.1 ± 4.9^a^ ^∗^	5.9 ± 1.8	6.2 ± 1.5^a^ ^∗^	2.2 ± 1.9^a^ ^∗^	3.0 ± 2.3^a^ ^∗^	31.4	57.9	69.8
EF400	84.8 ± 5.3^a^ ^∗∗^	3.6 ± 1.2^a^ ^∗∗^	4.0 ± 1.3^a^ ^∗∗^	1.7 ± 2.0^a^ ^∗∗^	2.0 ± 1.3^a^ ^∗∗^	58.1	44.7	46.5
AF100	73.9 ± 1.9^a^ ^∗^	5.5 ± 3.5^a^ ^∗^	6.3 ± 2.2^a^ ^∗^	2.5 ± 1.5^a^ ^∗^	2.8 ± 2.7^a^ ^∗^	36.1	65.8	65.1
AF200	90.6 ± 5.5^a^ ^∗∗^	4.3 ± 1.1^a^ ^∗^	4.8 ± 1.6^a^ ^∗∗^	1.9 ± 0.9^a^ ^∗^	2.1 ± 1.2^a^ ^∗∗^	50.0	50.1	48.8
AF400	109.8 ± 2.6^a^ ^∗∗∗^	2.3 ± 0.7^a^ ^∗∗∗^	2.9 ± 1.3^a^ ^∗∗∗^	1.5 ± 0.7^a^ ^∗∗^	1.6 ± 0.9^a^ ^∗∗^	73.2	39.5	37.2

*Note:* Values are presented as mean ± SEM (*n* = 6). Statistical comparisons were carried out using one‐way ANOVA followed by Tukey′s post hoc test.

Abbreviations: % inhi., percent inhibition; AF, aqueous fraction; ATR5, atropine, 5 mg/kg; CARE, *C. aurea* crude extract; CF, chloroform fraction; EF, ethyl acetate fraction; min, minute; NC, negative control (distilled water, 10 mL/kg); No., number; Wt., weight; WTFO, weight of total fecal output; WWFO, weight of wet fecal output.

^a^Compared with the negative control.

^∗^ *p* < 0.05,  ^∗∗^ *p* < 0.01, and  ^∗∗∗^ *p* < 0.001.

#### 3.4.2. Castor Oil–Induced Enteropooling

**Figure 3:**
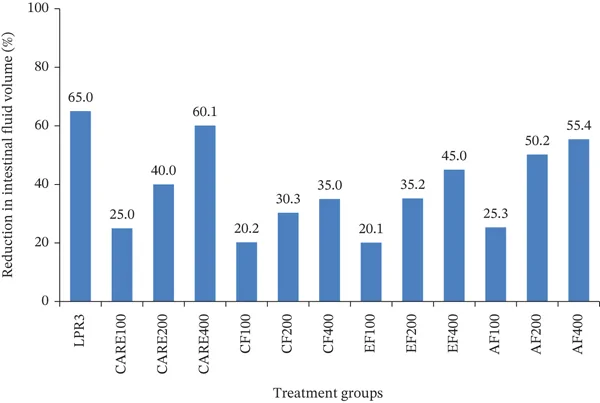
Percent reduction in intestinal fluid volume by *C. aurea* crude extract and solvent fractions in the castor oil‐induced enteropooling model in mice. Note: Mice were orally pretreated with vehicle (distilled water, 10 mL/kg), loperamide (LPR3; 3 mg/kg; positive control), the 80% methanolic crude extract of *C. aurea* (CARE; 100, 200, and 400 mg/kg), or the chloroform fraction (CF), ethyl acetate fraction (EF), and aqueous fraction (AF) at doses of 100, 200, and 400 mg/kg (*n* = 6 mice per group). Percent reduction in intestinal fluid volume was calculated relative to the negative control group.

The effect of the crude extract and solvent fractions on castor oil–induced enteropooling was evaluated by measuring intestinal content weight and volume (Table 4). CARE400 produced the greatest reduction, inhibiting weight (*p* < 0.001) and volume (*p* < 0.01) by 51.7% and 60.1%, respectively. Among the fractions, CF at 400 mg/kg dose caused a moderate but significant decrease (*p* < 0.05) of 31.0% in weight and 35.0% in volume. The EF also showed significant activity at 200 mg/kg (*p* < 0.05) and 400 mg/kg (*p* < 0.01), reducing both parameters. Similarly, the AF significantly decreased intestinal contents at 200 and 400 mg/kg, with the highest effect at the later dose (*p* < 0.001), accounting for 55.2% and 55.4% reductions, respectively (Figure 3).

**Table 4:** Effects of the crude extract and solvent fractions of the root of *C. aurea* on intestinal fluid accumulation in mice.

Group	Wt. of intestinal content (g)	Vol. of intestinal content (mL)	Reduction of wt. of intestinal contents (%)	Reduction of vol. of intestinal contents (%)
NC	2.9 ± 0.65	2.0 ± 0.46	—	—
LPR3	1.2 ± 0.91^a^ ^∗∗∗^	0.7 ± 0.34^a^ ^∗∗∗^	58.6	65.0
CARE100	2.6 ± 0.84	1.5 ± 0.41	10.3	25.0
CARE200	1.7 ± 0.70^a^ ^∗^	1.2 ± 0.22^a^ ^∗^	41.4	40.0
CARE400	1.4 ± 0.19^a^ ^∗∗∗^	0.8 ± 0.10^a^ ^∗∗^	51.7	60.1
CF100	2.7 ± 0.60	1.6 ± 0.25	6.9	20.2
CF200	2.5 ± 0.32	1.4 ± 0.71	13.8	30.3
CF400	2.0 ± 0.18^a^ ^∗^	1.3 ± 0.54^a^ ^∗^	31.0	35.0
EF100	2.5 ± 0.32	1.6 ± 0.15	13.7	20.1
EF200	2.2 ± 0.17^a^ ^∗^	1.3 ± 0.80^a^ ^∗^	24.1	35.2
EF400	1.7 ± 0.52^a^ ^∗∗^	1.1 ± 0.65^a^ ^∗∗^	41.4	45.0
AF100	2.3 ± 0.90^a^ ^∗^	1.5 ± 0.09^a^ ^∗^	20.7	25.3
AF200	1.6 ± 0.64^a^ ^∗∗^	1.0 ± 0.50^a^ ^∗∗^	44.8	50.2
AF400	1.3 ± 0.45^a^ ^∗∗∗^	0.9 ± 0.47^a^ ^∗∗∗^	55.2	55.4

*Note:* Findings are presented as mean ± SEM (*n* = 6). Statistical comparisons were conducted using one‐way ANOVA followed by Tukey′s post hoc test.

Abbreviations: AF, aqueous fraction; ATR5, atropine, 5 mg/kg; CARE, *C. aurea* crude extract; CF, chloroform fraction; EF, ethyl acetate fraction; g, gram; mL, milliliter; NC, negative control (distilled water, 10 mL/kg); Vol., volume; Wt., weight.

^a^Compared with the negative control.

^∗^ *p* < 0.05,  ^∗∗^ *p* < 0.01, and  ^∗∗∗^ *p* < 0.001.

#### 3.4.3. Castor Oil–Induced Antimotility Test

**Figure 4:**
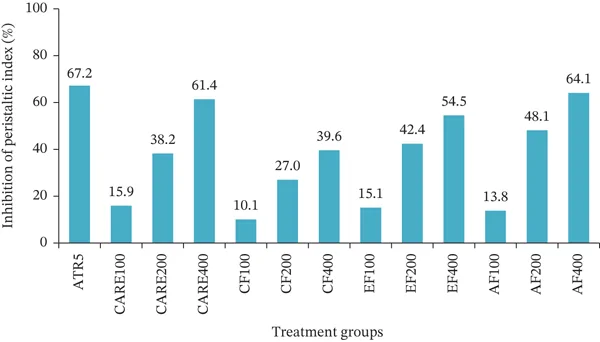
Percentage inhibition of the peristaltic index by *C. aurea* crude extract and solvent fractions in the gastrointestinal transit model in mice. Note: Mice were orally pretreated with vehicle (distilled water, 10 mL/kg), atropine sulfate (ATR5; 5 mg/kg; positive control, administered intraperitoneally), the 80% methanolic crude extract of *C. aurea* (CARE; 100, 200, and 400 mg/kg), or the chloroform fraction (CF), ethyl acetate fraction (EF), and aqueous fraction (AF) at doses of 100, 200, and 400 mg/kg (*n* = 6 mice per group). Percent inhibition of the peristaltic index was calculated relative to the negative control group.

The effect of the crude extract and its solvent fractions on intestinal transit was assessed using the charcoal meal test (Table 5). At all test doses, CARE produced a dose‐dependent reduction in propulsion, with the 400 mg/kg dose highly significantly lowering the peristaltic index (*p* < 0.001) and achieving 61.4% inhibition. CF showed significant activity only at 400 mg/kg (*p* < 0.05). EF showed significant antimotility effects at 200 mg/kg (42.4% inhibition, *p* < 0.001) and 400 mg/kg (54.5% inhibition, *p* < 0.001). AF significantly reduced the peristaltic index at middle (200 mg/kg) and high (400 mg/kg) test doses, with the highest inhibition at 400 mg/kg (64.1%, *p* < 0.001), nearly comparable to atropine (Figure 4).

**Table 5:** Effects of the crude extract and solvent fractions of the root of *C. aurea* on gastrointestinal transit in mice.

Group	Length of small intestine (cm)	St. travelled by charcoal meal (cm)	PI (%)	% inhibition
NC	60.5 ± 0.9	32.4 ± 1.1	53.6 ± 1.8	—
ATR5	58.6 ± 0.7	10.3 ± 2.2^a^ ^∗∗∗^	17.6 ± 1.2^a^ ^∗∗∗^	67.2
CARE100	61.2 ± 1.1	27.6 ± 1.7^a^ ^∗^	45.1 ± 2.0	15.9
CARE200	59.5 ± 1.3	19.7 ± 2.8^a^ ^∗∗^	33.1 ± 2.6^a^ ^∗∗^	38.2
CARE400	57.4 ± 1.4	11.9 ± 1.5^a^ ^∗∗∗^	20.7 ± 0.9^a^ ^∗∗∗^	61.4
CF100	59.7 ± 1.5	28.8 ± 3.0	48.2 ± 3.1	10.1
CF200	57.8 ± 0.4	22.6 ± 2.5^a^ ^∗∗^	39.1 ± 1.4	27.0
CF400	58.3 ± 0.6	18.9 ± 1.0^a^ ^∗∗^	32.4 ± 1.9^a^ ^∗^	39.6
EF100	58.7 ± 1.2	26.7 ± 2.3	45.5 ± 2.9	15.1
EF200	59.9 ± 0.8	18.5 ± 1.4^a^ ^∗∗∗^	30.9 ± 3.4^a^ ^∗∗∗^	42.4
EF400	60.2 ± 2.5	14.7 ± 2.1^a^ ^∗∗∗^	24.4 ± 1.5^a^ ^∗∗∗^	54.5
AF100	56.9 ± 1.9	26.3 ± 0.9	46.2 ± 2.9	13.8
AF200	57.2 ± 1.7	15.9 ± 1.8^a^ ^∗∗∗^	27.8 ± 2.5^a^ ^∗∗∗^	48.1
AF400	58.0 ± 1.4	11.2 ± 2.0^a^ ^∗∗∗^	19.3 ± 1.7^a^ ^∗∗∗^	64.1

*Note:* Data are presented as mean ± SEM (*n* = 6). Statistical comparisons were performed using one‐way ANOVA followed by Tukey′s post hoc test.

Abbreviations: AF, aqueous fraction; ATR5, atropine, 5 mg/kg; CARE, *C. aurea* crude extract; CF, chloroform fraction; cm, centimeter; EF, ethyl acetate fraction; NC, negative control (distilled water, 10 mL/kg); PI, peristaltic index; St., distance.

^a^Compared with the negative control.

^∗^ *p* < 0.05,  ^∗∗^ *p* < 0.01, and  ^∗∗∗^ *p* < 0.001.

#### 3.4.4. In Vivo ADI

**Figure 5:**
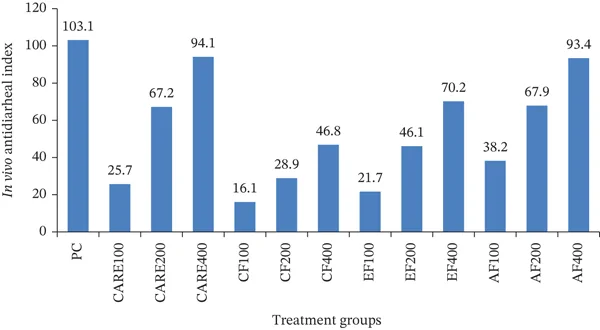
In vivo antidiarrheal index of *C. aurea* crude extract and solvent fractions in mice. Note: Groups evaluated (*n* = 6 mice per group) included the positive control (PC), the 80% methanolic crude extract of *C. aurea* (CARE; 100, 200, and 400 mg/kg), and the chloroform fraction (CF), ethyl acetate fraction (EF), and aqueous fraction (AF) at doses of 100, 200, and 400 mg/kg. Higher ADI values indicate greater overall in vivo antidiarrheal efficacy.

The ADI values for the treatment groups are presented in Table 6. Both the crude extract and solvent fractions of *C. aurea* demonstrated dose‐dependent antidiarrheal activity. Among the crude extract groups, CARE400 had the highest ADI (94.1), followed by CARE200 (67.2) and CARE100 (25.7). Among the fractions, AF showed the maximum effect, with AF400 (93.4) nearly equivalent to the positive control (103.1) (Figure 5).

**Table 6:** Effects of the crude extract and solvent fractions of the root of *C. aurea* on antidiarrheal indices in mice.

Group	Dfreq	Gmeq	Pfreq	ADI
NC	—	—	—	—
PC	205.9	68.2	77.9	103.1
CARE100	34.0	14.8	33.7	25.7
CARE200	123.2	39.2	62.8	67.2
CARE400	182.5	63.3	72.1	94.1
CF100	25.1	11.1	15.1	16.1
CF200	38.4	30.2	20.9	28.9
CF400	66.0	41.7	37.2	46.8
EF100	29.3	17.6	19.8	21.7
EF200	72.7	42.9	31.4	46.1
EF400	108.9	54.6	58.1	70.2
AF100	82.1	18.8	36.1	38.2
AF200	123.2	50.9	50.0	67.9
AF400	170.4	65.4	73.2	93.4

Abbreviations: ADI, antidiarrheal index; AF, aqueous fraction; CARE, *C. aurea* root extract; CF, chloroform fraction; Dfreq, delay in defecation time; EF, ethyl acetate fraction; Gmeq, gut meal travel reduction; NC, negative control; PC, positive control; Pfreq, purging frequency.

## 4. Discussion

This study evaluated the traditional use of *C. aurea* root extracts for managing diarrhea using established murine models. Both the crude extract and its solvent fractions demonstrated significant, dose‐dependent antidiarrheal activity.

Methanol (80%) was employed as the extraction solvent due to its high efficiency in yielding a broad spectrum of bioactive compounds. Methanol also inhibits microbial proliferation during the extraction process [36], which further supports its selection in this study.

Extraction of *C. aurea* root powder yielded 16.6% crude extract. Subsequent fractionation produced the highest yield in the AF (42.4%), followed by the EF (33.0%) and CF (24.6%). The predominance of the Aqueous fraction (AF) indicates a high abundance of polar phytochemicals such as flavonoids, tannins, and saponins, which likely contribute to the plant’s antidiarrheal effect.

Phytochemical screening revealed the presence of alkaloids, tannins, flavonoids, saponins, steroids, terpenoids, and phenols, all of which are well validated for their gastrointestinal modulatory effects [37]. Specifically, tannins enhance mucosal integrity and reduce fluid accumulation via protein precipitation and chloride channel inhibition [38, 39]. Flavonoids, phenols, and terpenoids attenuate intestinal hypersecretion, oxidative stress, and smooth muscle contractility, largely through the modulation of prostaglandin pathways [40, 41]. Additionally, alkaloids and saponins inhibit intestinal motility by blocking enteric cholinergic signaling or calcium influx [42]. Thus, the antidiarrheal activity of the extract could be linked with the reported pharmacological properties of these constituents, further supported by the notable absence of laxative anthraquinones and glycosides [43].

The acute oral toxicity study showed that the crude extract was well tolerated at the limit dose of 2000 mg/kg, with no observed mortality or overt clinical signs of toxicity. These findings point out that the median lethal dose (LD_50_) is greater than 2000 mg/kg, consistent with previous reports on *C. aurea* [44], and provide preliminary evidence of acute oral tolerability [45]. However, the absence of toxicity following a single dose does not establish general systemic safety.

In the castor oil–induced diarrhea model, the crude extract demonstrated a significant, dose‐dependent antidiarrheal effect. The marked delay in diarrhea onset, alongside reductions in the number and weight of wet feces, points to notable antisecretory and antimotility mechanisms. Because ricinoleic acid (the active metabolite of castor oil) promotes hypersecretion and intestinal motility through local inflammation and prostaglandin E_2_ release [46], the extract′s action could plausibly involve the attenuation of prostaglandin‐mediated inflammatory pathways. These findings agree with previous reports on related antidiarrheal medicinal plants, including *V. amygdalina* [33], *B. antidysenterica* [34], and *M. addat* [47]. Fractional analysis revealed that the AF (400 mg/kg) produced the highest antidiarrheal response, followed by the EF, whereas the CF was the least active. This activity gradient corresponds directly to solvent polarity and constituent distribution. The polar AF and semi‐polar EF are enriched in flavonoids, tannins, saponins, and alkaloids known for antidiarrheal efficacy, whereas the CF lacks these key bioactive metabolites.

To elucidate the potential mechanisms underlying the antidiarrheal activity of *C. aurea*, the extracts were further evaluated using enteropooling and gastrointestinal motility models. The enteropooling assay measures inhibition of intraluminal fluid accumulation [48]. In this model, both the crude extract and the AF produced substantial, dose‐dependent reductions in intestinal fluid volume and weight, peaking at 400 mg/kg, with the EF demonstrating slightly lower efficacy. These results indicate that the crude extract and its active fractions exert marked antisecretory effects. Prostaglandins and nitric oxide signaling pathways have been reported to enhance intestinal fluid secretion and alter mucosal permeability during diarrhea [49, 50]. Therefore, the observed antisecretory activity of the extract may be related to modulation of these secretory mediators and preservation of mucosal integrity.

To evaluate gastrointestinal transit, the antimotility effects of the crude extract and its fractions were assessed using the charcoal meal test. The crude extract, AF, and EF produced significant, dose‐dependent inhibition of intestinal peristalsis. At 400 mg/kg, the crude extract inhibited the peristaltic index by 61.4%, comparable to that of the reference drug atropine (67.2%). At the same dose, the AF exhibited greater inhibition (64.1%) than the crude extract, which may reflect enrichment of bioactive phytochemicals during fractionation. Suppression of intestinal transit prolongs mucosal contact time, thereby promoting fluid and electrolyte reabsorption. Cholinergic stimulation enhances intestinal peristalsis, an effect counteracted by anticholinergic agents such as atropine [51]. Consequently, the observed antimotility activity might be attributable to anticholinergic‐like effects or smooth muscle relaxation. Bioactive constituents identified in the extracts, such as flavonoids, alkaloids, and tannins, are well known to exhibit spasmolytic properties [52], likely accounting for the observed inhibition of intestinal transit.

The present findings demonstrate that the 80% methanolic root extract and solvent fractions of *C. aurea* possess significant, dose‐dependent antidiarrheal activity. These findings are consistent with previous reports on *C. aurea* leaves and seeds evaluated using similar bioassays [23, 24], extending the evidence of pharmacological activity to the roots. Similar antidiarrheal effects are well documented for *C. macrostachyus* [53], *M. stenopetala* [54], *C. myricoides* [36], *C. africana* [55], *W. somnifera* [10], and *O. lamiifolium* [56]. Comparable activities have also been reported in other Fabaceae taxa, such as *A. lebbeck* [57], *B. monosperma* [58], *C. fistula* [59], *C. costata* [60] and *I. spicata* [61]. However, these comparative evaluations should be interpreted cautiously, as variations in doses, solvents, animal strains, test protocols, and phytochemical profiles could affect the observed effects. Although the detected bioactive chemicals may contribute to the observed activity, the specific active constituents and underlying cellular mechanisms were not validated and require further investigation.

The ADI evaluates overall antidiarrheal efficacy by integrating parameters from the castor oil–induced diarrhea and intestinal motility models [62], with higher values indicating greater effectiveness [63]. In this study, all extracts exhibited a dose‐dependent increase in ADI, peaking at the maximum dose of 400 mg/kg. Notably, the AF consistently yielded the highest ADI among the solvent fractions across all doses, suggesting superior efficacy. As shown in Table 2, the broad range of phytochemicals present in the AF likely contributes to its superior efficacy.

Both male and female mice were included to assess the antidiarrheal activity of the crude extract and solvent fractions of *C. aurea* roots. Because this work focused on broad pharmacological screening rather than sex‐specific variations, data from both sexes were pooled for analysis. Nevertheless, given that biological sex can influence drug responses through variations in hormonal status, gastrointestinal physiology, and drug metabolism, sex‐specific differences cannot be ruled out. Future studies with balanced sex allocation and adequately powered subgroup analyses are warranted to investigate these potential variations.

Despite the promising findings, certain limitations should be acknowledged. First, the active secondary metabolites responsible for the observed effects were not isolated, quantified, or structurally characterized using techniques such as HPLC or LC–MS/MS. Second, pharmacokinetic characteristics, including oral absorption, systemic bioavailability, and tissue distribution, were not investigated. Third, although the in vivo models exhibited antidiarrheal activity, the underlying mechanisms were not experimentally verified through ex vivo isolated organ bath studies, tissue electrophysiological assessments, or specific receptor‐binding assays. Fourth, safety evaluation was limited to acute oral toxicity testing. Therefore, further subacute and chronic toxicity studies assessing organ weights, hematological and biochemical parameters, and histopathological changes are warranted. Finally, these findings were based on preclinical models; thus, clinical trials are required to confirm efficacy and safety in humans. Addressing these limitations through bioassay‐guided compound isolation, mechanistic investigations, comprehensive toxicological evaluations, and clinical trials should be prioritized in future research.

## 5. Conclusions

The 80% methanolic root extract and its solvent fractions of *C. aurea* exhibited significant, dose‐dependent antidiarrheal activity in mice, providing experimental validation for the traditional use of the plant′s root. These antidiarrheal effects may stem from antisecretory activity and the modulation of gastrointestinal motility, potentially mediated by the bioactive phytochemicals identified in the extract. However, these proposed mechanisms warrant further validation through dedicated ex vivo, molecular, and targeted functional assays.

**Nomenclature**
ADI: antidiarrheal index
AF: aqueous fraction
CARE: *C. aurea* root extract
CF: chloroform fraction
EF: ethyl acetate fraction
GI: gastrointestinal
OECD: Organization for Economic Cooperation and Development
WTFO: weight of total fecal output
WWFO: weight of wet fecal output.

## Author Contributions

All authors contributed significantly to this work. Y.A.F.: conceptualization, data collection, writing—review and editing. M.M.Z.: formal analysis, data curation, supervision. T.A.M.: conceptualization, methodology. A.B.K.: validation, writing—original draft. W.S.Z.: data collection, writing—review and editing. F.D.B.: methodology, supervision, formal analysis. M.A.A.: conceptualization, validation. All authors agree to take full responsibility for the content and findings of this study.

## Funding

No funding was received for this manuscript.

## Ethics Statement

The samples were collected from private land with full permission from the landowner. The species is not classified as protected, and no special government license was required for collection. Ethical approval for the study was obtained from the College of Health Sciences Ethical Review Committee of Debre Tabor University (Reference No. CHS/105/2024). All experimental procedures involving animals were conducted in accordance with the ARRIVE guidelines.

## Conflicts of Interest

The authors declare no conflicts of interest.

## Data Availability

Data analyzed or generated in this study can be accessed by contacting the corresponding author, subject to a reasonable request.
